# High Social Support System among Elderly in a Hilly District: A Descriptive Cross-sectional Study

**DOI:** 10.31729/jnma.7750

**Published:** 2022-10-31

**Authors:** Kalpana Paudel, Atit Tiwari

**Affiliations:** 1 Pokhara Nursing Campus, Institute of Medicine, Tribhuvan University, Pokhara, Kaski, Nepal; 2 Department of Psychiatry, B.P. Koirala Institute of Health Sciences, Dharan, Sunsari, Nepal

**Keywords:** *elderly*, *social support*, *social support system*

## Abstract

**Introduction:**

Social support is an important contributing factor that promotes the health of the elderly by providing them with positive experiences, socially satisfying roles, or the ability to cope with stressful situations. The objective of this research study is to find out the prevalence of a high social support system among the elderly in a Hilly district.

**Methods:**

A descriptive cross-sectional study was conducted among the elderly from a hilly district of Nepal. The ethical approval was taken from Ethical Review Board (Reference number: 3050). Data were collected from 20 May 2019 to August 2019. The elderly aged 60 years and above were the study population. A convenience sampling technique was used. The face-to-face interview was taken by using a structured questionnaire. Point estimate, and 95% Confidence Interval were calculated.

**Results:**

Out of 263 elderly people, 188 (71.48%) (66.02-76.94, 95% Confidence Interval) were provided with high support by their family members, friends, and significant others.

**Conclusions:**

The prevalence of high social support among the elderly was found to be higher when compared to similar studies conducted in similar settings.

## INTRODUCTION

Social support is a multifaceted concept including emotional support, tangible support, interaction or exchange support, and community support that improve physical and psychological well-being.^1,2^ It is a significant social determinant of health that has a stronger effect on health-promoting behaviour, selfefficacy and healthy ageing.^3-6^

Psychological distress in elders is associated with the lack of social support and somatic health problems. Nepalese communities are neighbourhood-based and religious-based that provide emotional and spiritual support to the elderly and also provide support for health care. But the prevalence of social support among Nepalese elderly is not entirely understood.^7^

This study aimed to find out the prevalence of a high social support system among the elderly in a Hilly district of Nepal.

## METHODS

A descriptive cross-sectional study was conducted among the elderly in the Kaski district. Data collection was done from 20 May 2019 to August 2019 after getting ethical approval from the Ethical Review Borad of Nepal Health Research Council (Reference number: 3050) and written permission from the concerned authority (District Health Office, Kaski). Respondents of age 60 years and above were included in the study. A convenience sampling technique was used.

The sample size was calculated using the following formula:


$$\begin{array}{ll} \text{n} & ={\text{Z}}^{2}\times \frac{\text{p}\times \text{q}}{{\text{e}}^{\text{2}}}\\ & =1.96^{2}\times \frac{0.50\times 0.50}{0.07^{2}}\\ & =196 \end{array}$$

Where,

n= minimum required sample size
Z= 1.96 at 95% Confidence Interval (CI)
p= prevalence of high social support taken as 50% for maximum sample size
q= 1-p
e= margin of error, 7%

The required sample size was 195. However, 263 elderly people were enrolled in the study. The social support system was measured by a standard tool called the Multidimensional Scale of Perceived Social Support (MMSPSS). The MSPSS measures perceived social support from three sources: family, friends, and significant others. This Likert scale consists of 12 items ranging from 1 (very strongly disagree) to 7 (very strongly agree). A mean scale score ranging from 1 to 2.9 could be considered low support; a score of 3 to 5 could be considered moderate support; a score from 5.1 to 7 could be considered high support.^8^ The Nepali version of the MSPSS was used. This scale was previously translated into the Nepali language, and its validity has been assessed in the Nepalese population.^9^ The interview was taken with each respondent in a friendly environment in their own home setting.

Collected data was analyzed in IBM SPSS Statistics version 16.0. Point estimate and 95% CI were calculated.

## RESULTS

**Figure 1:**
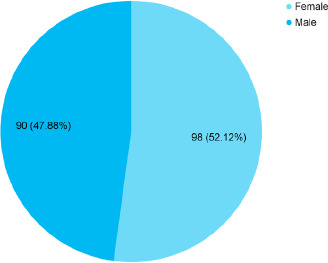
Gender-wise distribution of the elderly with a high level of social support (n= 188).

Among 263 elderly, 188 (71.48%) (66.02-76.94, 95% CI) were provided with high social support. Among them, 102 (54.26%) elderly were 70 years and above and 98 (52.12%) were females (Figure 1).

More than half of the married elderly 99 (52.65%) had a high level of social support. A high level of social support was found among the elderly who were literate 164 (87.24%) and had financial support 186 (98.93%). Regarding occupation, 100 (53.20%) were unemployed. More than two third 149 (79.25%) elderly who had comorbidity were provided with high support (Table 1).

**Table 1:** Socio-demographic characteristics (n= 188).

Characteristics	n (%)
**Age group**	
60-69 years	86 (45.74)
70 years and above	102 (54.26)
**Marital status**	
Married	99 (52.65)
Single	89 (47.35)
**Education**	
Illiterate	24 (12.76)
Literate	164 (87.24)
**Occupation**	
Employed	88 (46.80)
Unemployed	100 (53.20)
**Family type**	
Nuclear	39 (20.75)
Joint	149 (79.25)
Financial support	186 (98.93)
Co-morbidities	149 (79.25)

## DISCUSSION

Social support for the elderly is an important contributor to healthy ageing.^10^ There is suggestive evidence that poor social support may characterize a vulnerable situation to the psychological well-being of older people.^11^ Social support from friends or others is important because it helps the elderly to provide a better opportunity to be understood and share experiences.^12^ Our study depicted that 71.48% were provided with high support which is similar to the study conducted on community-dwelling older adults in the United States i.e., 62% reported having many close social ties in their lives.^13^ Whereas our study contradicts another study conducted in the United States which showed low perceived social support among the older-old people.^14^ Likewise, the finding of this study also differs from the studies done in Brazil^15^ and China^16^ which showed a moderate level of social support. The variation in the finding of the studies might be because of different tools in assessing social support.

In this study, elderly who were 70 years and above had a high level of social support and females received high social support than males which differed from the study done in India which showed that social support was low among the age groups of more than 80 years and females were provided with low social support.^17^

As this was a descriptive cross-sectional study, it studied the study population at one point in time. Such a design does not examine longitudinal fluctuations in the psychological well-being of the elderly. The limitation of this study is that it was conducted with elderly people with similar sociocultural characteristics. Finding has limited generalizability as the data were gathered from a single district only.

## CONCLUSIONS

The prevalence of high social support among the elderly was found to be higher when compared to similar studies conducted in similar settings. In our study, females had high social support than males. Social factors such as high support from family, friends, and social groups could be important predictors important predictors of psychological wellbeing among the elderly and further studies are recommended. Thus, elderly-friendly social support systems should be generated to increase the quality of life of elderly people.
